# Integration of mental health care in private not-for-profit health centres in Guinea, West Africa: a systemic entry point towards the delivery of more patient-centred care?

**DOI:** 10.1186/s12913-020-4914-3

**Published:** 2020-01-28

**Authors:** Abdoulaye Sow, Jeroen De Man, Myriam De Spiegelaere, Veerle Vanlerberghe, Bart Criel

**Affiliations:** 1 Fraternité Médicale Guinée, Conakry, Guinea; 2 Institute of Tropical Medicine, Antwerp, Belgium; 3 Université Libre de Bruxelles, Brussels, Belgium

**Keywords:** Mental health, Quality of care, Patient-centred care, Patient participation, Not-for-profit health centres, Guinea

## Abstract

**Background:**

Patient-centred care is an essential component of quality of health care. We hypothesize that integration of a mental health care package into versatile first-line health care services can strengthen patient participation, an important dimension of patient-centred care. The objective of this study is to analyse whether consultations conducted by providers in facilities that integrated mental health care score higher in terms of patient participation.

**Methods:**

This study was conducted in Guinea in 12 not-for-profit health centres, 4 of which had integrated a mental health care package (MH+) and 8 had not (MH-). The study involved 450 general curative consultations (175 in MH+ and 275 in MH- centres), conducted by 18 care providers (7 in MH+ and 11 in MH- centres). Patients were interviewed after the consultation on how they perceived their involvement in the consultation, using the Patient Participation Scale (PPS). The providers completed a self-administered questionnaire on their perception of patient’s involvement in the consultation. We compared scores of the PPS between MH+ and MH- facilities and between patients and providers.

**Results:**

The mean PPS score was 24.21 and 22.54 in MH+ and MH- health centres, respectively. Participation scores depended on both care providers and the health centres they work in. The patients consulting an MH+ centre were scoring higher on patient participation score than the ones of an MH- centre (adjusted odds ratio of 4.06 with a 95% CI of 1.17–14.10, *p* = 0.03). All care providers agreed they understood the patients’ concerns, and patients shared this view. All patients agreed they wanted to be involved in the decision-making concerning their treatment; providers, however, were reluctant to do so.

**Conclusion:**

Integrating a mental health care package into versatile first-line health services can promote more patient-centred care.

## Background

Quality of care is an area of major concern for health systems managers, and is of a complex and multidimensional nature [1]. One of its components is patient-centred care (PCC), which relates to the interaction between health providers and patients. PCC means that the patient, as a person, is at the centre of care [2]. Mead & Bower give a comprehensive overview of the PCC concept based on 5 dimensions: the biopsychosocial perspective, the “patient as a person”, the sharing of power and responsibility, the therapeutic alliance, and the “care provider as a person” [3]. Appropriate patient-provider communication is an important aspect of PCC, is pivotal for patient self-management and has been shown to benefit their health [4–6]. However, Jaffré & Olivier de Sardan found that in reality, in most West African settings, the interaction between patients and care providers is brief and instrumental, with little room for dialogue and mutual understanding [7].

In the consultation setting, PCC requires the provider to actively listen to the patients and their family and to actively involve them in decision-making [3]. The result of this process is a jointly established and mutually agreed treatment plan [8, 9]. In the field of mental health care, the “person-centred” approach has generally been interpreted as a holistic approach, taking into account the patient’s unique experience, culture and needs [4, 10–12]. Adequate mental health care requires an appropriate interaction between providers and patients, including genuine patient participation.

The “Montréal model” [13], based on a partnership between patients and care providers, is based on recognising the experiential knowledge of patients, resulting from living with their illness, and on the additional scientific knowledge of the care provider. Together it is part of a continuum of patient engagement. Carman et al. define a 4-step theoretical framework of this engagement: 1. Information: patients receive information (diagnosis and treatment); 2. Consultation: patients are consulted about their preferences in relation to treatment; 3. Cooperation: treatment decisions take into account therapeutic preferences and 4. Partnership: patients are followed-up in the self-management to help them control their health problem [14].

In low- and middle-income countries, HIV/AIDS programmes often succeeded in adopting a patient-centred approach to care, but in many instances with limited integration into the general health services system [15–17]. This may result in creating “islands” of high quality care for HIV/AIDS patients, while the care offered to patients consulting for other health problems remains problematic [18]. Hence the strong relevance for health care delivery of trying to properly integrate specialised programmes in general health services [19, 20]. It is precisely from this perspective that our study investigates the impact of the integration of a mental health care package into first line health services on quality of care, and more in particular on patient participation in the consultation.

### Study context

Mental health services are poorly developed in sub-Saharan Africa, despite the increasing disease burden mental disorders are causing [21]. Notwithstanding the overwhelming evidence of the need for mental care, a remarkable deficit of providers for mental disorder service delivery persists in the continent [22]. This is not different in Guinea where there is virtually no offer of mental health care within the modern health care delivery system [23].

Primary health care in Guinea is essentially publicly organised. There are but a few not-for-profit primary health centres in Guinea, mainly concentrated in large cities. The secular NGO *Fraternité Médicale Guinée* (FMG) is one of the most important non-governmental actors in the Guinean health system and is operating several not-for-profit primary health centers. It was created 25 years ago by a group of young Guinean medical practitioners with the aim to offer comprehensive primary care at newly created first line health facilities, with, at least initially, a strong urban focus. These FMG health centres take care of the population of their catchment area and give special attention to marginalised population groups such as the mentally ill, sex workers, orphans, vulnerable children and travellers (lorry drivers, fishermen, merchants). To an important extent, they can provide these services with resources coming from so-called vertical programmes, or from specific projects.

Mental health was integrated in three of these not-for-profit FMG-run health centres in the late 1990s in the frame of the *Santé Mentale en Milieu Ouvert Africain* (SaMOA) project implemented by FMG [24]. SaMOA mobilised general practitioners, paramedics and community health workers of those three centres to dispense care and support to the mentally ill, knowing that none of these health professionals had received any substantial training in mental health during their undergraduate education, except for a shallow introduction to the subject. In a “first wave” of the program, these health care staff were trained in mental health issues via joint consultations, case studies, teamwork and remote support from specialists (psychiatrists, psychologists and social workers) from a Belgian mental health centre. Resources were mobilised to implement this programme and to train health staff, supply medication to the facilities, develop teamwork, involve communities in the care of patients and, at the same time, strengthen the functioning of these centres. The approach aimed to overcome fragmentation of services and enhance integration of care [18].

Between 2006 and 2012, the provision of mental health care in these three versatile first line healthcare facilities was extended to other not-for-profit health centres and to one public health centre. The training of what was now called the “second wave” was based on workshops and seminars. Successive evaluations of the mental health services integration in these healthcare facilities [25] led to the hypothesis that care providers who look after the mentally ill have a better interaction with patients who attend out-patient consultations for a wide variety of other health problems [26].

To test this hypothesis, qualitative research was undertaken among care providers who were trained in mental health and who offer mental health care at the front line (working in facilities henceforth labelled MH+), and those without any such specific post-graduate training in mental health who do not offer such care (working in facilities henceforth labelled MH-). The effect of integrating mental health care on providers’ attitude towards mental health and on the adoption of a patient-centred approach was analysed in five Guinean not-for-profit health centres [27]. Providers who offered mental health services had overcome their fears of mental health patients and developed more positive attitudes towards this group of patients. This positive effect was more pronounced among providers trained in the “first wave”. Care providers who offered mental health care had adopted a more patient-centred approach. The integration of mental health care/services is not the only factor explaining these changes. Other important factors are firstly the in-situ training (i.e. joint consultations, teamwork and community action) in which the emotional needs of care providers are considered, proposing a patient-centred role model; and secondly the characteristic of health care facilities, in particular their organisational culture, more specifically the non-bureaucratic context of one of the not-for profit, community oriented centres with a stable and qualified team consisting of staff trained during the “first-wave” [27].

Building further on this evidence, the present study seeks to investigate whether the approaches and attitudes acquired in the management of mental health problems actually ‘trickle down’ into the general care for patients that visit first-line services for a broad range of health problems. More specifically, we aim to test if routine out-patient consultations, conducted in health centres that had integrated mental health care score higher in terms of patient participation.

## Methods

### Description of the study site

**Fig. 1:**
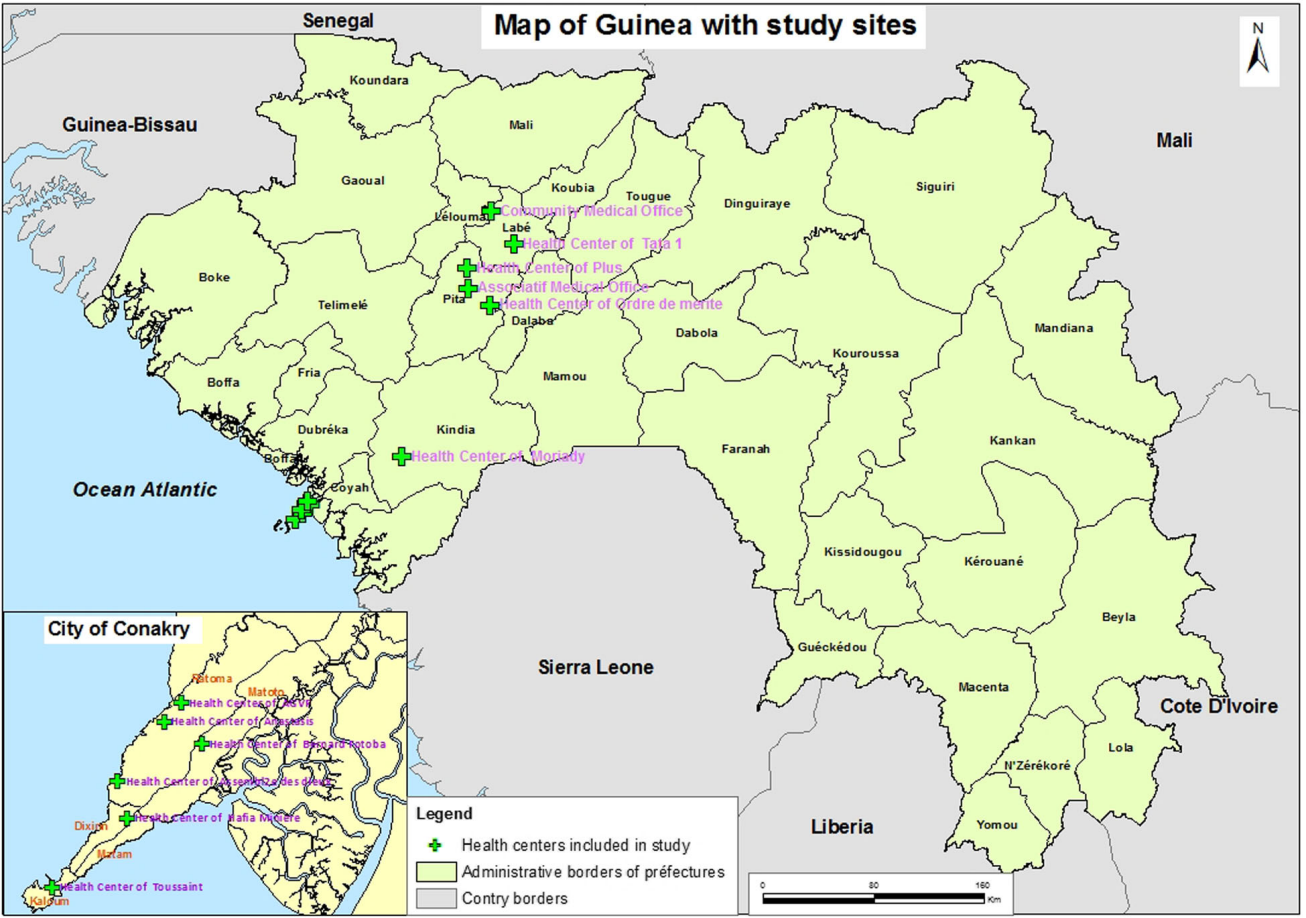
The map was constructed by the first author AS, with the help of a Guinean cartographer, using the ArcGIS software available for free on line

The study was conducted in 3 areas that have both not-for-profit MH+ and MH- health centres (Fig. 1).

### Recruitment of participants

12 health centres were selected among not-for-profit health facilities with similar administrative status, funding patterns and operations. Four of these 12 facilities had integrated a mental health package in their routine offer, eight had not. We chose not to include public or private-for-profit facilities in our study because they had either not integrated mental health in their package of activities or had an organizational culture and a way of functioning substantially different from the selected facilities.

Most of these 12 selected health centres are run by doctors and they are integrated in the local district health systems. In these 12 health centres, we included *all* providers in charge of the curative consultations in the out-patient department on a daily basis: eventually, this added up to 18 health workers (7 in MH+ facilities of which 5 doctors, 1 nurse-practitioner and 1 social worker; and 11 in MH- facilities of which 9 doctors and 2 nurse-practitioners). Our sample size (450 patients) was calculated to demonstrate a 50% satisfaction rate among participants about their involvement in the consultation process, with a precision of 5%. A sample of at least 175 inclusions in each group allows to detect a difference of 15% (at alpha 5% and beta 80%). In order to have a balanced representation per care provider, we opted to include an equal number, namely 25, of consultations per care provider resulting in 175 consultations for MH+ and 275 for MH- group (ratio MH−/MH+ of 1.5).

### Data collection and entry

The study is based on a two-tier data collection method: (a) exit interviews with patients consulting for primary curative care using the Patient Participation Scale (PPS) [12], and (b) a self-administered questionnaire based on the same scale for the providers who conducted these consultations. The following data were also collected: 1. patient’s demographics (age, gender and level of education); 2. whether the patient or an accompanying person responded; 3. whether the patient was accompanied by one or more relatives; 4. the demographic characteristics of the health care provider (age, sex, years of experience and professional qualification); and 5. the duration of the consultation. Patients were included independently of their health problem or their demographic characteristics. In the more frequented health centres, every 3rd consultation was included in the sample; in less frequented facilities all patients were interviewed. Based on this recruitment procedure, patients were randomly selected until a number of 25 patients was reached. The interviews with the 450 patients were conducted by a public health physician - the same for all interviews - in the vernacular of the patient and of the accompanying person. Double data entry was performed to minimize encoding errors. The interviewer was briefed and told that the purpose of the study was to investigate patient participation during out-patient consultations in a range of not-for-profit health centres. He was however not informed (kept ‘blind’) that eventually MH+ and MH- health facilities would be compared.

### Description of the data collection tool

The PPS tool [12] has been used in various studies to assess patient participation [28, 29]. It includes 6 questions that probe the extent to which patients feel that they have been able to effectively participate in the decision-making process about their health problem and treatment. The tool was adapted so as to facilitate understanding by study participants. More specifically, three questions (i.e. Q7–9) were added to the PPS tool to assess overall satisfaction and the patient’s preferred level of involvement in treatment decision-making (active or passive). The adapted tool was then translated into the two main local languages (Soussou and Poular; the Malinké language being only very rarely spoken) through a 3-step process: translation, counter-translation and drafting of a provisional version based on the comparison of the translations against the original version. This was subsequently pre-tested on 25 patients at a health centre not participating in the study to make sure the issues were understood correctly by potential study participants and to check the interview time. After modifications, a new version was tested on 15 patients in another health centre not participating in the study. The final questions are presented in Additional file 1. Responses were categorised using a Likert scale (from 1 to 5): (1) not at all, (2) no, (3) I do not know (neither agree nor disagree), (4) somewhat and (5) a lot. After the last consultation, a self-administered questionnaire was completed by the care providers who conducted the consultations. This questionnaire was also based on the PPS, but took the perspective of the provider (see Additional file 2). Testing and translation of this questionnaire followed the same procedures as the version for patients.

### Data analysis

Patient demographics (age, sex, education level), characteristics of the respondent - i.e. patients or their caretaker/companion) and duration of consultation were analysed using descriptive statistics. Question 7 was excluded from the analyses because 79% of patients did not answer this question, probably because it was seen as either not applicable or as a repeat of question 4. In order to evaluate the internal consistency of the 6 PPS questions (Q1–6), the McDonald’s total omega and hierarchical omega coefficients were calculated. A global participation score was created by summing all scores of the PPS items. A bivariate analysis between the variables of the health centres, providers, MH+ or MH- clusters on the one hand, and the variable ‘participation score’ on the other hand was then conducted. Multiple regression was conducted with the continuous variable ‘participation score’ as the dependent variable, and the independent variables that were significantly associated with the dependent variable. To control for the fact that patients were clustered by different providers and to account for non-normality, we used a ‘Generalised Estimated Equations (GEE)’ model for the analyses.

The answers to the questions were also analysed separately. Since participants’ scores were not distributed normally, they were regrouped in a dichotomous variable with the following categories: “agree” (initial answers options 4 and 5) and “disagree” (initial answers options 1 and 2). Answer option 3 “I do not know (unresolved opinion)” was considered neutral and was ignored in this part of the analysis. The proportion of participants who “agreed” to the different questions were compared between MH+ and MH- health centres, among the different providers, and between patients and providers. These proportions are also graphically represented to allow for a visual comparison of potential differences as recommended by Hirsh et al. [29].

The effect of integration of mental health was assessed by multi-level logistic regression for each of the questions (except for question 8). Demographic characteristics of patients that may have influenced patient-provider interaction, such as age, sex, and level of education were included as independent variables in this model. Providers were considered as a random effect. The model did not include the identity of the respondent (i.e. the patient or their caretaker/companion; the latter in case of consultations of under-fives) since no significant association was found between this particular feature and the responses. We could not make an analysis of question 6 of the PPS because the model did not converge, even when increasing the number of iterations and changing the model’s starting value. The intra-class correlation coefficient was calculated to estimate the extent to which the random variable or the care provider characteristics (age, gender, education) explained the variance of the calculated results. The analyses were carried out using SPSS and R software. For the multilevel analyses, the “glmer” function of the “lme4” package [30] in R and the GEE function in SPSS were used.

The study was approved by the National Commission on Ethics of Health Research (CNERS) of Guinea (N° 010/CNRS/17). The participants were informed about the purpose and the potential risks of the study before the start of the interview and freely agreed to participate. All data collected can be accessed by contacting the first author and principal investigator of the research.

## Results

### Characteristics of the study participants

The 450 consultations included 442 patients (2% did not consent), and all 18 care providers completed the self-assessment form.

#### Characteristics of study participants

The characteristics (sex, age, level of education, whether the patient came alone or was accompanied at the consultation, whether the patient responded personally or via the accompanying person) did not differ significantly between MH+ and MH- groups. However, the proportion of children aged 6–17 was higher in MH+ than in MH- facilities (see Table 1).

**Table 1:** Patients’ characteristics

Characteristics		MH+		MH-		OR
		*Number*	*%*	*Number*	*%*	
*Sex*	Female	101	57,7	135	50,6	REF
	Male	74	42,3	132	49,4	0.75
*Age*	0–5 years old	44	25,1	82	30,7	REF
	6–17 years old	45	25,7	36	13,5	2.3^a^
	18–44 years old	59	33,7	104	39,0	1
	> 45 years old	27	15,4	45	16,9	1.1
*Level of education*	No schooling	78	44,6	134	50,2	REF
	Primary school	63	36,0	71	26,6	1.5
	Secondary school or higher	34	19,4	62	23,2	0.9
*Manner of presentation*	Patient came alone	60	34,3	99	37,1	REF
	With a companion	91	52,0	147	55,1	1
	With more than one companion	24	13,7	21	7,9	1.9
*Respondent*	Patient	84	48,0	149	55,8	REF
	Companion	91	52,0	118	44,2	1.4
	Total	175		267		

*REF* reference group, *OR* Odds Ratio

^a^Statistical significance (threshold 0.05)

#### Care providers and consultation characteristics

A total of 18 care providers was consulted by 450 patients in 12 health facilities. Seven providers, including 5 doctors, 1 nurse and 1 social worker (a retrained biologist) carried out the consultations in the MH+ centres. In the MH- centres, the consultations were conducted by 11 providers: 9 doctors and 2 nurses. The median duration of a consultation was 7 min in both facilities, but with 6 min as percentile 25 and 11 min as percentile 75 in MH+ versus 5 min as percentile 25 and 9 min as percentile 75 in MH- facilities (*p* = 0.013, Mann-Whitney-Wilcoxon test).

### Analysis of the overall perception of patients of their involvement in the care process

The average participation score is 23.20 (with an average of 3.87 per question) and a standard deviation (SD) of 4.21 (range 11 to 30). Participation scores among providers range from 19.12 to 26.96 (*p* < 0.001). Among health facilities scores range from 20.49 to 26.96 (p < 0.001). The participation score varies according to the health centre and to the consulting provider. The mean participation score is 24.21 (SD 3.75) and 22.54 (SD 4.36) for respectively MH+ and MH- health centres (see Table 2). For an increase of one unit in the participation score, the odds to have consulted a MH+ provider is five times higher than the odds to have consulted a MH- provider (crude odds ratio of 5.29 with a 95% CI of 1.35–20.78, *p* = 0.02). In general, care providers working in MH- health centres obtain relatively low scores in comparison to those in MH+ centres. However, there are exceptions: a doctor running a solo practice in a rural area and working in a MH- centre obtained the highest score. The internal consistency of the 6 PPS questions is satisfactory with a total McDonald omega coefficient of 0.83 and a hierarchical omega coefficient of 0.58. In the multivariable analysis, patients who visited a MH+ centre have a significantly higher participation score than those who consulted in a MH- centre (adjusted OR of 4.06 with a 95% CI of 1.17–14.10; *p* = 0.03).

**Table 2:** Perception of patients of their involvement in the consultation process: Multivariable analysis based on a linear ‘Generalised Estimate Equation’ model

Characteristics	Mean score (SD) of participation	Crude OR (95% CI)	Adjusted OR (95% CI)
Mental health			
- MH+	24.21 (3.75)	5.29 (1.35–20.78) *	4.06 (1.17–14.10) *
- MH-	22.54 (4.36)	1	1
Patient’s age (continuous variable)	NA	1.00 (0.97–1.03)	
Patient’s gender			
- Male	23.11 (4.15)	0.96 (0.52–1.74)	
- Female	23.29 (4.27)	1	
Patient’s level of education			
- University	22.90 (5.20)	0.95 (0.08–10.68)	
- Secondary	23.23 (4.39)	1.26 (0.49–3.23)	
- Primary	22.79 (4.04)	0.47 (0.20–1.09)	
- No schooling	23.49 (4.11)	1	
Interview language:			
- Soussou	21.60 (4.29)	0.47 (0.10–2.29)	
- Poular	23.82 (3.77)	0.28 (0.08–0.96)	
- Malinke	21.16 (4.53)	0.49 (0.07–3.61)	
- French	23.24 (4.56)	1	
Age of the care provider (continuous variable)	NA	1.03 (0.93–1.14)	
Number of years of experience of the care provider (continuous variable)	NA	1.01 (0.93–1.10)	
Professional level of care provider:			
- Doctor	23.25 (4.33)	1.33 (0.23–7.60)	
- Non-medical	22.96 (3.56)	1	
Duration of the consultation (continuous variable)	N/A	1.22 (1.08–1.37) *	1.21 (1.08–1.37) *

*N/A* Not Applicable

*value p < 0.05

### Analysis of the different dimensions of patients’ perception of their involvement in the care process

**Fig. 2:**
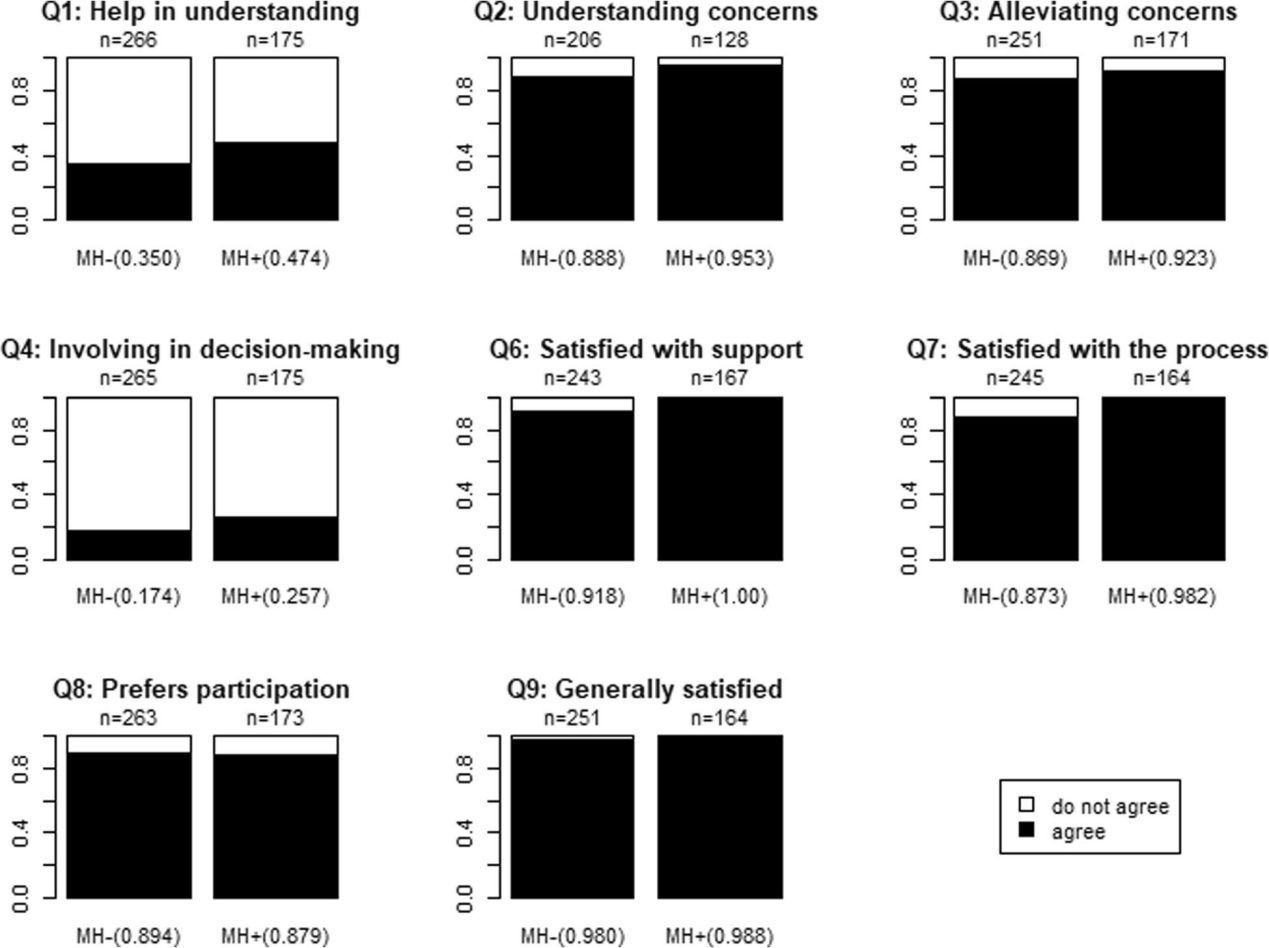
MH + = health facilities with mental health package, MH- = health facilities without mental health package

Out of the 8 exit interview questions, patients disagree most on questions 1 and 4 (Fig. 2). The answers reflect the extent to which the patients feel supported by the care provider in participating in the consultation. The answers to question 8 suggest that patients want to be more actively involved in decision-making about their treatment. Paradoxically, the answers to questions 6 and 7 are generally positive, suggesting that participants are satisfied with their level of involvement. The answers to questions 2 and 3 indicate that patients generally believe that care providers adequately understand and address their concerns, and question 9 indicates that they are generally satisfied with the consultation process. The results presented in Fig. 2 indicate a difference in favour of facilities that offer an integrated mental health package, with the exception of questions 8 and 9.

### Comparison of care providers

**Fig. 3:**
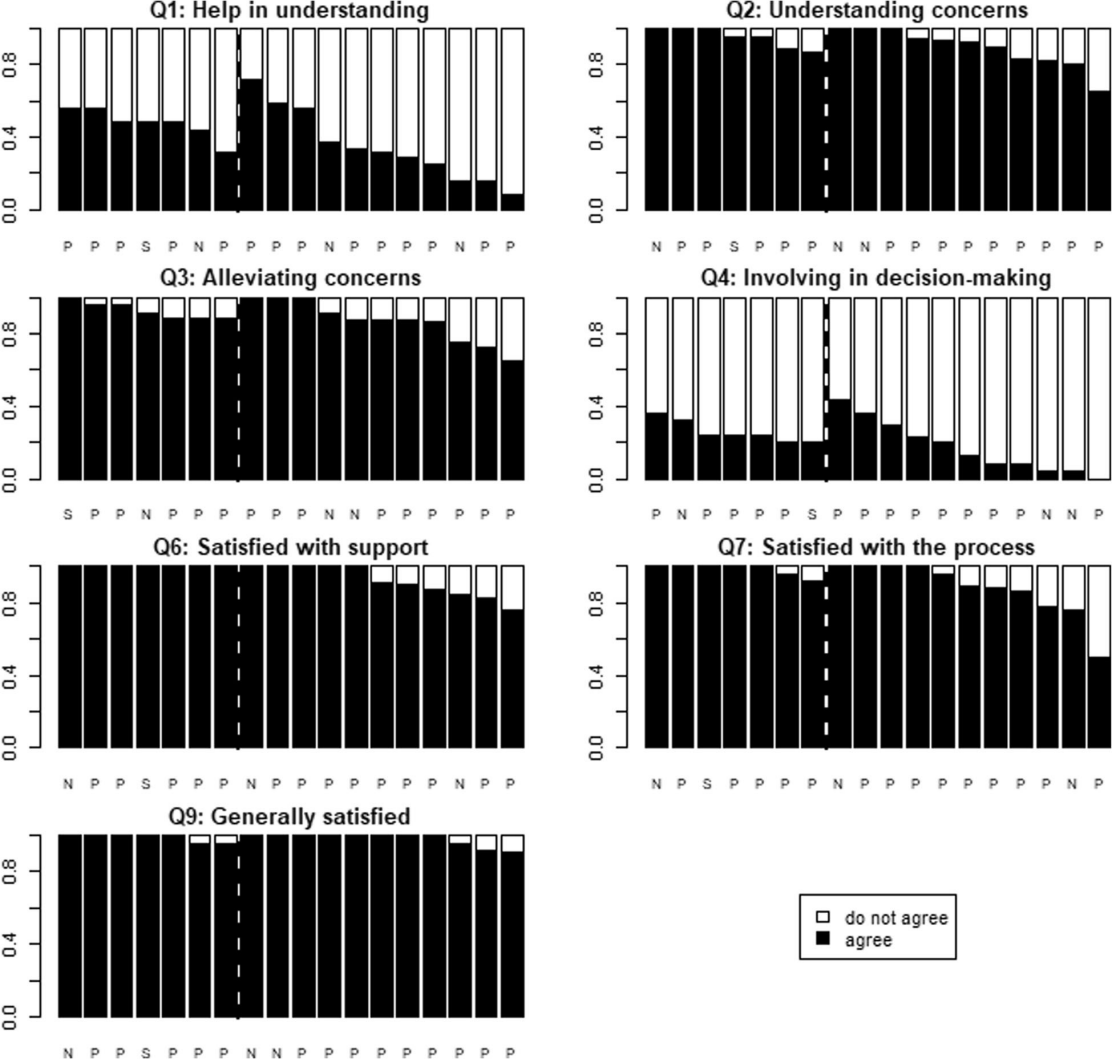
N = Nurse, P = Physician, S = Social worker

Figure 3 shows the proportion of positive responses given by the patients in relation to the type of care provider (MH+ centres on the left side of the dotted line and MH- centres on the right). We note substantial variance in the proportion of positive responses across care providers for all questions except for question 9. The intraclass correlation coefficient (ICCs in Table 3), obtained from a multilevel multivariable analysis, indicates the variance in the scores because of different care providers. For questions 1 to 4, this coefficient lies between 4 and 8%. This indicates that the care provider plays a relatively important role in the score of these questions. For question 7, this is relatively less, but still considerable. There is no indication that the type of provider - physician or non-physician - has an influence on the outcome of the separate questions (Fig. 3). However, as non-physicians are in the minority (4/18), the scope of our study does not allow us to take a clear stance on this issue.

**Table 3:** Results of the multi-level logistic regression

	Q1: Comprehension support		Q2: Understanding of the concerns		Q3: Worries are alleviated		Q4: Participation in decision-making		Q7: Satisfaction about the process		Q9: General satisfaction	
Fixed effects	est	IC 95	est	IC 95	est	IC 95	est	IC 95	Est	IC 95	est	IC 95
Intercept	0.68	0.39–1.20	8.80	3.03–25.58	6.90	3.02–15.75	0.27	0.14–0.53	13.66	3.30–56.46	70.57	7.43–670.20
MH+	1.82	0.95–3.49	2.68	0.77–9.31	1.79	0.70–4.59	1.93	0.91–4.09	8.69	1.18–64.02	1.78	0.24–12.90
Age	1.00	0.99–1.01	1.00	0.98–1.02	1.00	0.98–1.02	0.99	0.98–1.00	1.02	1.00–1.04	1.01	0.97–1.05
Sex	0.91	0.61–1.37	1.83	0.79–4.28	1.01	0.54–1.90	0.96	0.59–1.55	1.11	0.49–2.54	2.38	0.44–12.92
Education	0.70	0.46–1.05	0.72	0.31–1.66	1.23	0.64–2.34	0.90	0.55–1.47	0.42	0.16–1.08	0.41	0.07–2.28
Random effects	var	CCI	var	CCI	var	CCI	var	CCI	Var	CCI	var	CCI
Care providers	0.2868	0.084	0.6176	0.068	0.4375	0.055	0.3431	0.054	1.948	0.19	0.9483	0.014

*est* parameter estimate, *var* variance between facilities, *IC 95* 95% confidence interval, *ICC* intra-class correlation coefficient, *MH+* integrated mental health

### Comparison of perceptions of care providers and patients

**Fig. 4:**
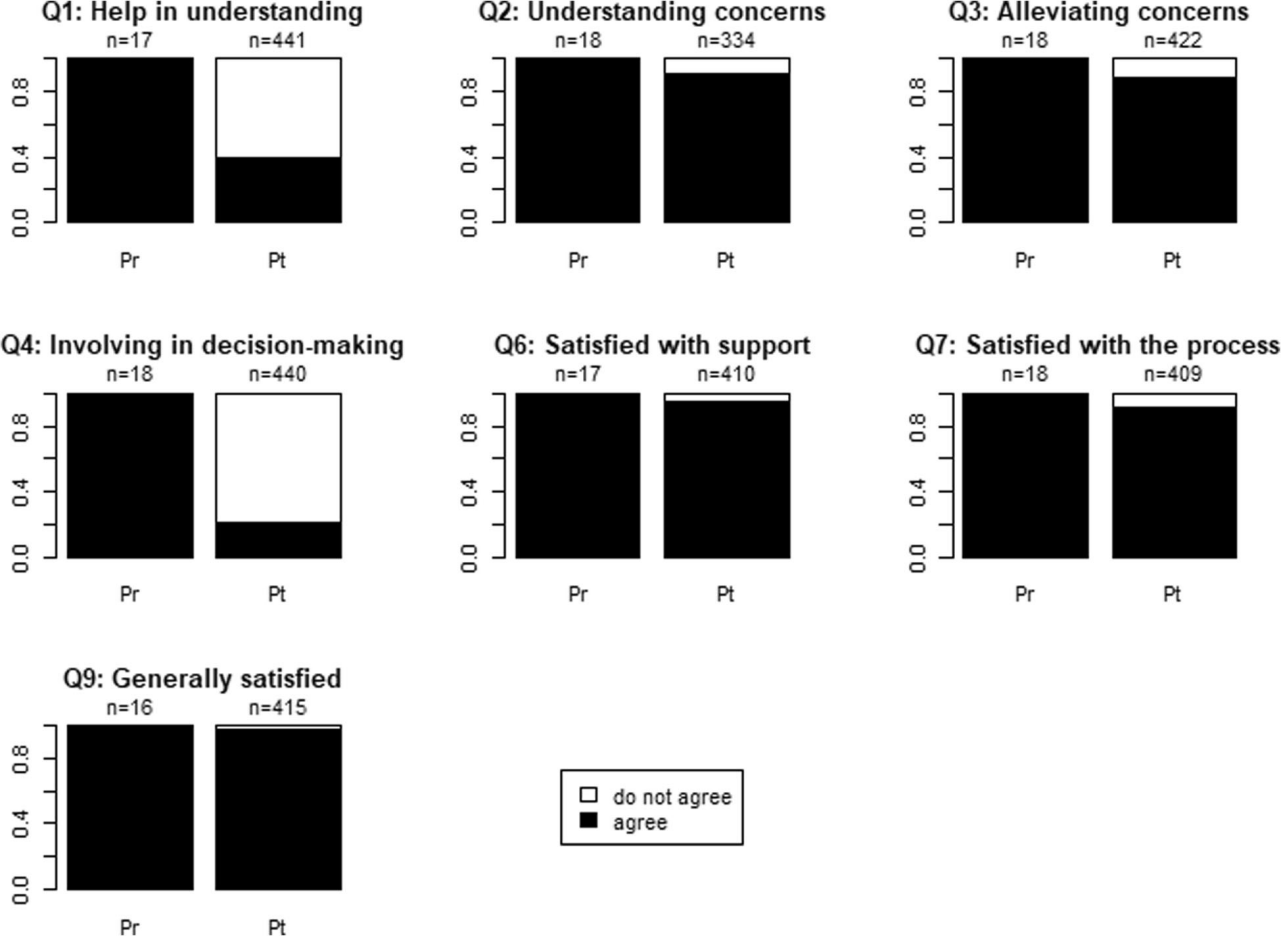
Pr = Provider, Pt = Patient

The scores that providers (MH+ and MH-) give themselves are all positive (Fig. 4). However, there are significant differences between this self-assessment and the perception of patients regarding the help they receive in understanding the information related to their illness (question 1) and regarding their involvement in the treatment decision-making (question 4). The agreement between patients is high for the other questions.

## Discussion

To our knowledge, our study is unique in comparing two types of health centres (MH+ and MH-) in terms of patient-provider communication during primary curative consultations. Our study analyses patient satisfaction with their participation during the consultation and self-assessment of providers of their interaction with patients. Based on the data collected from 442 patients and the 18 providers that were consulted, the results of our research indicate that patient satisfaction concerning their involvement in the consultation process is generally higher (*p* = 0.03) for those who visit a MH+ facility (adjusted OR 4.06 with IC 95%: 1.17–14.10). The perception of participation depends on the health centre the patients consulted, the individual care provider who received them, the MH+ or MH- orientation of the services and the duration of the consultation. Our findings show that the integration of mental health alone does not on its own explain the enhanced quality of care in the MH+ health centres through better patient participation in the consultation process. The characteristics of the care facility also matter.

Care providers of MH+ and MH- are all satisfied with the way they involve patients in the decision-making process regarding their therapeutic trajectory. They feel that they properly understand and express the concerns of patients. This observation is shared by the patients. However, our results also indicate that patients want to be involved in the curative consultation process, even though their satisfaction with their involvement is in contradiction with this wish. This discrepancy may be explained by the patient’s understanding of participation and/or the existence of a social desirability bias that makes patients respond positively to the question pertaining to satisfaction. Our data do not provide a clear-cut answer and further research would be needed to explore the type and modalities of the involvement the patients desire (and under what circumstances and for which health problem).

Our results suggest that the quality of communication is better in health centres that have trained mental health care providers. The statistically significant difference in the average participation score confirms our hypothesis. When the answers to the questions are taken individually, we observe no significant differences in some of them. This study did not take account of some other important factors that may have contributed to the “trickling down” of PCC, such as the nature and intensity of the training received by care providers (“first wave” versus “second wave” trainees) and the years of experience in caring for the mentally ill [27].

Nevertheless, our results suggest a difference in the perception of patients consulting in health centres that offer a mental health package. The higher patients’ scoring of participation in health centres that offer a mental health care package, could be explained by the management model for mental illness that implies a strong interaction between the patients, their family, their entourage and their care providers [6]. This approach promotes a higher extent of patient-centred care. Care providers in MH+ centres may have appropriated this care model and adopted it in the general outpatient consultations, beyond patients presenting with a mental health problem. There is in fact a commonality between the approach to mental care provision and patient-centred care, including a level of empathy, listening, respect for the person, long-term follow-up, etc. [31]. Consequently, changes in the care providers’ attitudes towards patients with a mental disorder could have led to a subtle, natural, organic, even unconscious change in their approach to patients presenting with other problems [27]. These changes could be a result of the way in which training was conducted during the mental health integration process [32]. However, there may be other reasons for this change, such as the specific characteristics of care providers, the organisational culture of the facilities in which they work, and the way in which mental health has been integrated. Indeed, the integration of mental health into the framework of the SaMOA project may have encouraged care providers to question themselves in their daily practice and could have triggered them to find a better balance between the needs for respect and listening to the patient and the need for their own protection as a care provider.

The analysis of patient characteristics in this study, particularly gender, level of education and age, indicate that there is no significant difference between the two populations except for patients in the 6–17 age group where we find a significantly higher number of children in MH+ than in MH- facilities (Odds ratio of 2.3, *p* = 0.003). There is no clear reason for this difference other than a particular mix of patients at a given point in time. On the other hand, we have no evidence to suggest that the difference in this particular subgroup would have affected our interpretation of the results and our conclusions.

Our study points to a discrepancy between the providers’ perception of their own behaviour and patients’ perceptions of their own involvement in the consultation. It is possible to conclude that patients are satisfied despite their low involvement, but as discussed above, other reasons could explain the patient’s satisfaction score.

According to research conducted in other contexts, patients tend to feel that they should not interfere in the decisions taken by the care provider, as the latter is seen to be autonomous in the decision-making process. The care providers for their part considers themselves to be the experts and the only ones able to decide what is right or wrong in the treatment of the patient [7, 33]. If this would be the case in our study population, improving communication would require a change in the balance of power between care provider and patient [6].

A qualitative analysis focusing on the more specific issues of relationships between the patient and the provider (their experience, the type of pathology suffered by the patient, the care provider’s level of and approach to training, ..) would provide us with additional information on the challenges of this interaction in the context of primary health care in Guinea.

### Limitations

The present study has a number of limitations. Our data concern not-for-profit health centres, which only account for a small number of first line health care facilities in Guinea. Public health centres, although more numerous (410/450) where not included in this study as they have a different mode of operation. Our results can therefore not be extrapolated to the other health centre categories in Guinea. Our study was based on a series of questions inspired by a validated tool in high-income countries [12, 29]. Although we adapted it to the local context, the statistical distribution of the responses did not allow us to analyse the results of the 5 Likert scale answer options. The PPS tool indeed borrowed from Western family medicine and may to some extent apply to the practice of primary health care in Guinea, but its use as a measurement tool needs to be adapted to take account of contextual parameters that we may not have fully identified. The Likert scale also presents shortcomings in its interpretation by patients [34]. Guinean patients often are unprepared to comment on the care provider’s attitude. Patients, perhaps out of fear of “retaliation”, may prefer not to score the care providers’ attitudes towards them. The type of pathology (diagnosis) suffered by the patients was not taken into consideration in our study and could also have influenced their answers. The limited number of health centres with integrated mental health is low, which obviously limits the statistical power.

## Conclusion

Our study indicates that integrating mental health into first-line health care services can lead to a more patient-centred approach. Nonetheless, a comprehensive and multi-pronged approach is a prerequisite for improving quality of care. Our analysis confirms that improving PCC requires a change of factors at different levels of the system, including the health facility, the providers and the patients.

## Supplementary information

**Additional file 1.** Adapted Patient Participation Scale (PPS) with questions submitted to the patients.

**Additional file 2.** Self-assessment of care providers: list of self-administered questions.

## Data Availability

The datasets used and/or analysed during the current study available from the corresponding author on reasonable request.

## Abbreviations

CNERS: National Commission on Ethics of Health Research
FMG: Fraternité Médicale Guinée
GEE: General Estimated Equations
ICC: Intraclass Correlation Coefficient
OR: Odds Ratio
PCC: Patient-Centred Care
PPS: Patient Participation Scale
SaMOA: Santé Mentale en Milieu Ouvert Africain
SD: Standard Deviation
