# Supplementary material for: Integration of mental health care in private not-for-profit health centres in Guinea, West Africa: a systemic entry point towards the delivery of more patient-centred care?
Source: BMC Health Serv Res. 2020 Jan 28;20:61. doi: 10.1186/s12913-020-4914-3 (PMC6986146; doi:10.1186/s12913-020-4914-3)
Supplement: Supplementary file 1 — Additional file 1. Adapted Patient Participation Scale (PPS) with questions submitted to the patients. [file 12913_2020_4914_MOESM1_ESM.docx]

Additional file 1: Adapted Patient Participation Scale (PPS) with questions submitted to the patients

| Q1. Did your doctor help you understand the information related to your illness? |
| --- |
| Q2. Does your doctor understand your concerns/your worries? |
| Q3. Did your doctor alleviate your concerns/your worries? |
| Q4. Did your doctor involve you in the therapeutic decision-making process? |
| Q5. Are you satisfied with the doctor's contribution to your treatment decision? |
| Q6. Are you satisfied with the way your treatment was discussed and decided? |
| Q7. Are you glad that you were involved in the decision-making about your treatment? |
| Q8. Would you have liked/or have wanted to be involved in the decision-making regarding your treatment? |
| Q9. In general, are you satisfied with the way your consultation was handled? |
