# Supplementary material for: Integration of mental health care in private not-for-profit health centres in Guinea, West Africa: a systemic entry point towards the delivery of more patient-centred care?
Source: BMC Health Serv Res. 2020 Jan 28;20:61. doi: 10.1186/s12913-020-4914-3 (PMC6986146; doi:10.1186/s12913-020-4914-3)
Supplement: Supplementary file 2 — Additional file 2. Self-assessment of care providers: list of self-administered questions. [file 12913_2020_4914_MOESM2_ESM.docx]

Additional file 2. Self-assessment of care providers: list of self-administered questions

| Q1. I help my patients understand the information related to their illness/health problem |
| --- |
| Q2. I understand my patient’s concerns |
| Q3. I respond adequately to my patient’s concerns/worries |
| Q4. I involve the patients in therapeutic decision-making |
| Q5. I am satisfied with the way the treatment is discussed and decided with my patients |
| Q6. I am satisfied with the result of my patients’ involvement in therapeutic decision-making |
| Q7. I am satisfied with my input in the decision-making process about the treatment of my patients |
| Q8. I am satisfied with the way I handle the consultations |
